# Carbon stocks and accumulation rates in Red Sea seagrass meadows

**DOI:** 10.1038/s41598-018-33182-8

**Published:** 2018-10-09

**Authors:** Oscar Serrano, Hanan Almahasheer, Carlos M. Duarte, Xabier Irigoien

**Affiliations:** 1 School of Science, Centre for Marine Ecosystems Research, Edith Cowan University, 270 Joondalup Drive, Joondalup, 6027 Australia; 2 Department of Biology, College of Science, Imam Abdulrahman Bin Faisal University (IAU), Dammam, 31441-1982 Saudi Arabia; 3 King Abdullah University of Science and Technology (KAUST), Red Sea Research Center, Thuwal, 23955-6900 Saudi Arabia; 4 AZTI - Marine Research, Herrera Kaia, Portualdea z/g –, 20110 Pasaia, (Gipuzkoa) Spain; 5 IKERBASQUE, Basque Foundation for Science, Bilbao, Spain

## Abstract

Seagrasses play an important role in climate change mitigation and adaptation, acting as natural CO_2_ sinks and buffering the impacts of rising sea level. However, global estimates of organic carbon (C_org_) stocks, accumulation rates and seafloor elevation rates in seagrasses are limited to a few regions, thus potentially biasing global estimates. Here we assessed the extent of soil C_org_ stocks and accumulation rates in seagrass meadows (*Thalassia hemprichii*, *Enhalus acoroides*, *Halophila stipulacea*, *Thalassodendrum ciliatum* and *Halodule uninervis*) from Saudi Arabia. We estimated that seagrasses store 3.4 ± 0.3 kg C_org_ m^−2^ in 1 m-thick soil deposits, accumulated at 6.8 ± 1.7 g C_org_ m^−2^ yr^−1^ over the last 500 to 2,000 years. The extreme conditions in the Red Sea, such as nutrient limitation reducing seagrass growth rates and high temperature increasing soil respiration rates, may explain their relative low C_org_ storage compared to temperate meadows. Differences in soil C_org_ storage among habitats (i.e. location and species composition) are mainly related to the contribution of seagrass detritus to the soil C_org_ pool, fluxes of C_org_ from adjacent mangrove and tidal marsh ecosystems into seagrass meadows, and the amount of fine sediment particles. Seagrasses sequester annually around 0.8% of CO_2_ emissions from fossil-fuels by Saudi Arabia, while buffering the impacts of sea level rise. This study contributes data from understudied regions to a growing dataset on seagrass carbon stocks and sequestration rates and further evidences that even small seagrass species store C_org_ in coastal areas.

## Introduction

Seagrasses occupy only 0.1% of the ocean surface but are considered one of the largest carbon sinks worldwide^1–4^. Unlike terrestrial ecosystems, which store organic carbon (C_org_) mainly in living biomass, C_org_ stocks in seagrass meadows are mainly found in their soils, where it can accumulate over millennia^5,6^. Given their ability to capture and retain C_org_ and elevate the seafloor, seagrass meadows play a significant role in climate change mitigation and adaptation by sequestering greenhouse gases and buffering the impacts of rising sea levels^4^.

Seagrasses comprise more than 70 species of seagrasses^7^ that have a wide variation in traits including differences in primary production rates, below ground biomass, the recalcitrance of the C_org_ in their organs, and the ability of their canopies to trap allochthonous carbon^8^. Seagrasses occur across different depositional environments^9^, which results in highly variable soil C_org_ stocks among seagrass habitats (up to 18-fold^10^). Such differences are the result of the interactions among multiple biotic and abiotic factors (e.g. species composition, geomorphology, hydrodynamic energy and water depth) acting in the water column, canopy and the soils, that can affect rates of C_org_ deposition and accumulation over millenary time scales^11–15^. However, global estimates of C_org_ stocks in seagrass ecosystems have been calculated based on a very narrow data set, based on few species and habitats mainly from sites within the Mediterranean, Northern Atlantic, and eastern Indian Oceans^6,16^. Indeed, the paucity of C_org_ burial estimates in seagrass ecosystems is limiting our understanding of their carbon sequestration capacity and the assessment of differences in C_org_ storage across seagrass habitats. In order to improve global estimates of C_org_ stocks in seagrass meadows, research is needed that expands the current databases to include assessments in regions representing geomorphological and biological characteristics thus far underrepresented in the literature, such as arid regions.

The Red Sea, a tropical, arid region lacking riverine inputs, provides a case for a region currently underrepresented in the assessment of seagrass C_org_ stocks. Although C_org_ stocks within terrestrial ecosystems of the Red Sea arid bioregion are relatively low, coastal environments may serve as critical and important carbon sinks within this region^17^. While seagrass communities in the Red Sea are mainly dominated by opportunistic and small species^18^, even these small seagrasses have been documented to significantly enhance sediment stabilization and accumulation^19^ and potentially contributing to C_org_ storage^10,20^. Given that there are over 370 km^2^ of seagrasses in Saudi Arabia^18,21^, there is potential for these meadows to contain substantial stores of C_org_. This study aims to provide estimates of soil C_org_ stocks and accumulation rates in seagrass meadows from the Red Sea, thus contributing to achieve a more balanced representation of variation among seagrass habitats by considering underrepresented regions^10^, and to place our results within a larger context by comparing these data to estimates from global datasets.

We combine estimates of soil C_org_ density down to 1 m depth with soil chronologies derived from ^14^C age dating to estimate (a) C_org_ stocks within the top meter of soil, and (b) the accumulation rate of C_org_ over the last millennia. We also estimate the contribution of autochthonous and allochthonous sources to the seagrass soil C_org_ pool and determine soil grain-size composition to examine their relationships with soil C_org_ pools.

## Results

Soils in Red Sea seagrass meadows were mainly constituted of clay and silt particles (37 ± 0.7% on average), with a relatively high abundance of very fine sands (21 ± 0.4%) compared to fine sands (16 ± 0.4%), medium sands (12 ± 0.3%) and coarse sands (14 ± 0.7%) (Table 1). The average (±SE) dry bulk density in 1 m-thick Red Sea seagrass soils was 1.05 ± 0.01 g cm^−3^ and the average C_org_ concentration was 0.33 ± 0.01% and 3.05 ± 0.07 mg cm^−3^. The soil accretion rates in Red Sea seagrass meadows over centennial time scales ranged broadly, between 0.02 and 1.57 cm yr^−1^ (Table 2). In 1 m-thick soil deposits and over 500 to 2,000 years of accumulation, seagrass meadows accumulated, on average, 3.4 ± 0.3 kg C_org_ m^−2^ at a rate of 6.8 ± 1.7 g C_org_ m^−2^ yr^−1^ (Table 2).

**Table 1:** Average ± standard error (SE) of (a) dry bulk density (in g cm^−3^), organic carbon (C_org_) content (in % and mg cm^−3^), δ^13^C signatures of soil organic matter and (b) sediment grain-size content at Red Sea seagrass soil cores (for the total length of core sampled; see Supporting Information Table B for further details).

(a) Core ID	Seagrass spp.	Dry bulk density (g cm^−3^)			C_org_ (%)			C_org_ (mg cm^−3^)		δ^13^C (‰)	
		N	Mean	SE	N	Mean	SE	Mean	SE	Mean	SE
T1	*H. stipulacea*	117	1.26	0.01	68	0.24	0.02	2.98	0.17	−18.61	0.35
T2	*H. stipulacea*	17	1.18	0.04	17	0.24	0.02	2.96	0.40	−18.41	0.48
T3	*H. stipulacea*	20	1.05	0.02	20	0.27	0.02	2.82	0.21	−17.78	0.55
EC1	*T. hemprichii*	8	0.69	0.05	8	0.80	0.05	5.38	0.37	−11.95	0.64
EC2	*T. hemprichii*	42	0.64	0.03	31	0.72	0.03	4.45	0.15	−10.91	0.19
EC3	*T. hemprichii*	8	0.94	0.05	8	0.50	0.06	4.61	0.45	−11.45	0.10
EC4	*E. acoroides*	18	0.73	0.02	18	0.57	0.04	4.09	0.18	−11.63	0.19
EC5	*E. acoroides*	136	0.93	0.02	77	0.45	0.03	3.75	0.19	−13.08	0.20
EC6	*E. acoroides*	105	0.92	0.01	61	0.32	0.02	2.65	0.14	−13.22	0.08
EC7	*E. acoroides*	20	1.10	0.04	20	0.40	0.06	3.99	0.39	−11.31	0.31
EC8	*T. ciliatum*	7	0.96	0.03	7	0.16	0.04	1.52	0.40	−14.14	0.18
EC9	*T. ciliatum*	7	0.89	0.09	7	0.36	0.10	2.74	0.32	−14.82	0.48
EC10	*T. ciliatum*	47	1.05	0.03	33	0.21	0.03	2.40	0.31	−15.43	0.24
PR1	*T. ciliatum*	164	1.14	0.01	92	0.14	0.01	1.61	0.07	−23.00	0.19
PR2	*T. ciliatum*	13	0.91	0.03	13	0.95	0.24	9.03	2.33	−22.94	0.35
PR3	*T. ciliatum*	15	1.02	0.01	15	0.18	0.02	1.89	0.17	−22.47	0.56
PR4	*T. ciliatum*	16	1.00	0.02	16	0.24	0.04	2.31	0.33	−22.91	0.31
KA1	*H. uninervis*	120	1.35	0.01	70	0.17	0.01	2.26	0.13	−14.13	0.13
KA2	*H. uninervis*	18	1.13	0.02	18	0.22	0.01	2.52	0.13	−14.44	0.06
KA3	*H. uninervis*	7	0.93	0.04	7	0.26	0.02	2.37	0.09	−14.30	0.16
KA4	*H. stipulacea*	6	0.80	0.04	6	0.29	0.04	2.30	0.28	−13.97	0.11
KA5	*H. stipulacea*	10	0.95	0.06	10	0.38	0.05	3.48	0.35	−15.11	0.13
KA6	*H. stipulacea*	9	0.85	0.07	9	0.39	0.06	3.05	0.33	−15.28	0.25
KA7	*H. stipulacea*	60	0.96	0.02	40	0.37	0.02	3.33	0.14	−14.81	0.09
KA8	*H. stipulacea*	10	0.96	0.07	10	0.42	0.03	3.96	0.23	−13.92	0.10
KA9	*H. stipulacea*	56	0.86	0.02	38	0.45	0.01	3.76	0.12	−14.23	0.05
KA10	*H. uninervis*	9	0.94	0.06	9	0.44	0.03	4.02	0.21	−14.03	0.05
**Total**		**1065**	**1.05**	**0.01**	**728**	**0.33**	**0.01**	**3.05**	**0.07**	**−16.02**	**0.15**
**(b) Core ID**	**N**	**<0.063** **mm**		**>0.063 <0.125** **mm**		**>0.125 <0.25** **mm**		**>0.25 <0.5** **mm**		**>0.5 <1** **mm**	
		**Mean**	**SE**	**Mean**	**SE**	**Mean**	**SE**	**Mean**	**SE**	**Mean**	**SE**
T1	56	44.0	2.6	34.2	1.5	17.7	0.9	2.7	0.2	1.4	0.2
T2	13	44.2	3.8	35.7	2.1	17.9	1.5	1.6	0.3	0.5	0.2
T3	14	42.7	3.2	33.8	1.5	16.2	1.1	5.2	0.6	2.1	0.4
EC1	8	52.3	3.1	22.4	0.7	8.7	0.6	8.4	1.1	8.1	2.3
EC2	31	44.2	0.7	25.3	0.8	12.7	0.4	9.4	0.4	8.4	0.7
EC3	8	21.7	1.0	19.7	0.6	20.5	0.5	19.0	0.4	19.1	1.0
EC4	14	40.42	2.0	16.8	0.5	11.8	0.5	13.8	0.5	17.2	1.4
EC5	53	52.3	1.3	16.5	0.4	10.2	0.3	10.4	0.5	10.6	0.8
EC6	55	38.6	0.9	13.8	0.4	10.5	0.3	16.0	0.4	21.2	0.9
EC7	16	30.6	1.9	12.0	1.1	8.9	0.4	16.9	0.7	31.6	2.7
EC8	7	6.3	2.5	2.5	0.7	2.8	0.9	22.3	1.8	66.0	5.3
EC9	7	21.1	4.1	7.0	1.6	4.2	0.8	19.5	1.8	48.2	5.0
EC10	33	11.2	1.6	4.7	0.7	3.9	0.4	20.9	0.7	59.3	2.7
PR1	49	31.6	2.6	35.6	1.4	28.2	1.3	4.5	0.5	0.1	0.0
PR2	11	15.1	2.5	28.6	1.2	37.1	1.4	15.2	1.5	4.0	0.7
PR3	12	26.1	4.6	32.5	1.2	31.3	2.8	9.1	1.5	1.0	0.3
PR4	13	29.5	5.1	35.2	2.6	27.5	2.5	6.3	1.0	1.4	0.3
KA1	54	10.8	0.4	18.6	0.3	29.9	0.5	24.1	0.2	16.5	0.7
KA2	15	26.7	1.0	15.1	0.6	15.9	0.4	19.8	0.6	22.5	1.3
KA3	7	32.8	1.5	16.0	0.5	13.3	0.4	17.1	0.9	20.8	1.1
KA4	6	40.6	3.1	17.2	1.0	11.0	0.3	13.6	1.4	17.6	2.4
KA5	10	44.8	1.9	14.4	0.7	10.0	0.3	14.0	0.8	16.8	1.9
KA6	9	46.1	3.8	14.1	1.3	9.1	0.4	13.3	1.5	17.5	3.3
KA7	40	43.7	0.9	15.0	0.3	11.6	0.3	13.7	0.3	16.0	0.6
KA8	10	57.0	1.4	21.2	0.8	8.3	0.3	6.8	0.5	6.7	1.5
KA9	38	60.8	1.1	20.0	0.3	8.5	0.2	6.1	0.4	4.7	0.6
KA10	9	61.5	1.6	21.0	0.7	8.4	0.4	5.7	0.9	3.3	1.1
**Total**	**598**	**36.8**	**0.7**	**21.1**	**0.4**	**15.6**	**0.4**	**12.0**	**0.3**	**14.4**	**0.7**

The total number of data (N) provides an indication of the core length. Cores T1 to T3 were sampled at Thuwal Island, cores EC1 to EC10 at Economic city, cores PR1 to PR4 at Petro Rabigh and cores KA1 to KA10 at Khor Alkarar.

**Table 2:** Soil accretion rates (cm yr^−1^) and organic carbon (C_org_) stocks (kg m^−2^) and accumulation rates (g m^−2^ yr^−1^) in Central Red Sea seagrass meadows (in 1 m-thick soils).

Core ID	Location	Seagrass species	Soil accretion rates (cm yr^−1^)		Stock	Accumulation rates
			Mean	SE	(kg C_org_ m^−2^)	(g C_org_ m^−2^ yr^−1^)
T1	Thuwal	*H. stipulacea*	0.36	0.10	2.66	9.58
T2	Thuwal	*H. stipulacea*	n/a	n/a	2.94	n/a
T3	Thuwal	*H. stipulacea*	0.17	0.03	2.42	4.01
EC1	Economic city	*T. hemprichii*	0.40	n/a	5.39	21.57
EC2	Economic city	*T. hemprichii*	0.09	0.03	4.34	3.98
EC3	Economic city	*T. hemprichii*	0.10	n/a	4.62	4.41
EC4	Economic city	*E. acoroides*	0.10	0.01	4.10	4.25
EC5	Economic city	*E. acoroides*	0.02	0.01	4.17	0.81
EC6	Economic city	*E. acoroides*	0.05	0.00	2.60	1.29
EC7	Economic city	*E. acoroides*	0.08	0.01	4.24	3.20
EC8	Economic city	*T. ciliatum*	0.05	0.01	1.51	0.73
EC9	Economic city	*T. ciliatum*	0.06	0.02	2.73	1.70
EC10	Economic city	*T. ciliatum*	0.28	0.03	1.95	5.42
PR1	Petro Rabigh	*T. ciliatum*	n/a	n/a	1.96	n/a
PR2	Petro Rabigh	*T. ciliatum*	n/a	n/a	9.69	n/a
PR3	Petro Rabigh	*T. ciliatum*	1.06	n/a	2.43	25.74
PR4	Petro Rabigh	*T. ciliatum*	1.57	0.21	1.84	28.77
KA1	Khor Alkarar	*H. uninervis*	0.13	0.05	2.09	2.74
KA2	Khor Alkarar	*H. uninervis*	0.08	0.03	2.61	2.19
KA3	Khor Alkarar	*H. uninervis*	n/a	n/a	2.36	n/a
KA4	Khor Alkarar	*H. stipulacea*	0.06	n/a	2.26	1.39
KA5	Khor Alkarar	*H. stipulacea*	0.07	n/a	3.50	2.58
KA6	Khor Alkarar	*H. stipulacea*	n/a	n/a	3.09	n/a
KA7	Khor Alkarar	*H. stipulacea*	0.12	0.03	3.22	3.81
KA8	Khor Alkarar	*H. stipulacea*	0.27	0.02	3.93	10.81
KA9	Khor Alkarar	*H. stipulacea*	0.17	0.03	3.66	6.17
KA10	Khor Alkarar	*H. uninervis*	0.10	n/a	4.00	3.90
**Mean**			**0.24**	**0.04**	**3.35**	**6.77**

The C_org_ stocks were extrapolated to 1 m in 14 out of 27 cores studied (see methods section for further details). Mean ± SE values are reported. n/a indicates variables that were not measured.

**Figure 1:**
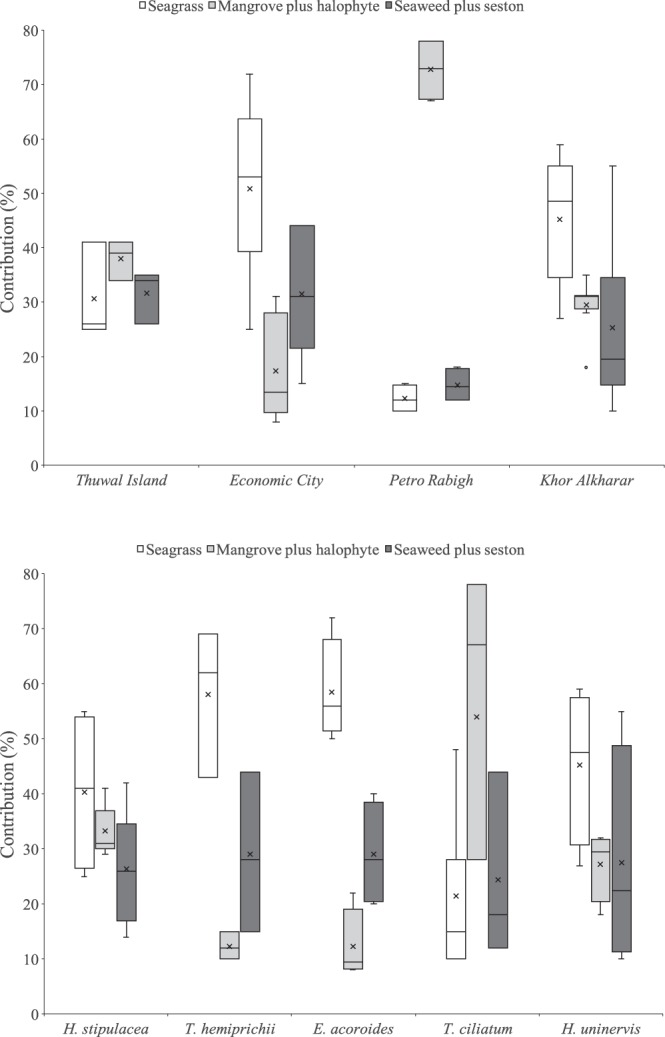
Boxplot showing the results of the isotopic mixing model (IsoSource software). Proportion (25%, 50% and 75% quantiles) of autochthonous (i.e. seagrass matter) and allochthonous (i.e. mangroves plus halophytes, seaweed and seston) C_org_ to the seagrass soil C_org_ pool (top 60 cm of the cores) based on study site (Economic City, Khor Alkharar, Petro Rabigh and Thuwal Island) and seagrass species (*Enhalus acoroides, Halodule uninervis, Halophila stipulacea, Thalassia hemprichii and Thalassodendrum ciliatum*).

The δ^13^C values of sedimentary organic matter in seagrass soils averaged −16 ± 0.2‰ (Table 1). The δ^13^C values of potential organic sources into seagrass soils collected at the four study sites are presented in Table 3. The mixing models applied indicated that seagrass detritus was the most important source of soil C_org_ in Red Sea seagrass meadows (41%), while mangrove plus halophytes and seaweed plus seston inputs were less important (32 and 27%, respectively; Fig. 1 and Supporting Information Table A).

**Table 3:** Mean (±SE) of isotopic carbon values (‰) of potential organic sources into seagrass soils collected at the four study sites.

Sources	Origin	Site	Species	N	δ^13^C (‰)
Seagrass	Autochthonous	Economic City	*E. acoroides*	6	−5.9 ± 0.1
			*T. hemprichii*	15	−6.6 ± 0.4
			*T. ciliatum*	12	−7.5 ± 0.4
		Khor Alkharar	*H. stipulacea*	18	−7.7 ± 0.2
			*H. uninervis*	9	−9.3 ± 0.5
		Petro Rabigh	*T. ciliatum*	12	−11.1 ± 0.2
		Thuwal Island	*H. stipulacea*	6	−7.5 ± 0.4
Mangrove & halophytes	Allochthonous	Economic City		39	−26.6 ± 0.2
		Khor Alkharar		42	−25.3 ± 0.6
		Petro Rabigh		24	−24.3 ± 0.9
		Thuwal Island		39	−26.1 ± 0.2
Seaweed		Economic City		3	−7.6 ± 0.6
		Khor Alkharar		18	−12.2 ± 0.8
		Petro Rabigh		n/a	n/a
		Thuwal Island		27	−13.7 ± 0.7
Seston		Economic City		3	−3.8 ± 2.1
		Khor Alkharar		3	−11.1 ± 2.5
		Petro Rabigh		3	−17.0 ± 0.9
		Thuwal Island		3	−13.5 ± 1.3

N indicates the number of samples analyzed. n/a indicates variables that were not measured. Mean ± SE values are reported.

**Figure 2:**
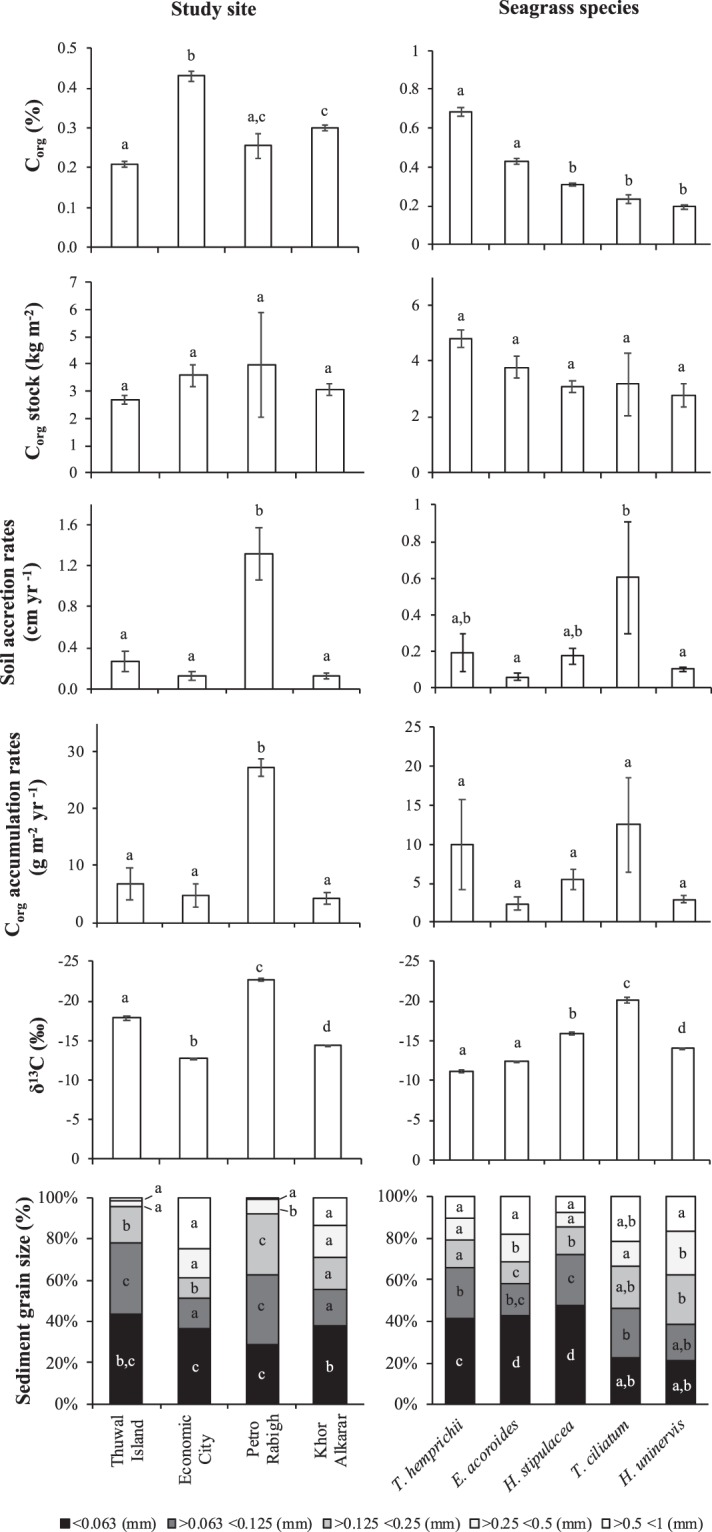
Soil accretion rates (cm yr^−1^) and organic carbon (C_org_) content (%), stocks (kg m^−2^ in 1 m-thick soils; extrapolated when necessary, see methods) and accumulation rates (g m^−2^ yr^−1^), stable carbon isotope signatures of soil organic matter (δ^13^C; in ‰) and sediment grain-size fractions in Central Red Sea seagrass meadows based on study site (Economic City, Khor Alkharar, Petro Rabigh and Thuwal Island) and seagrass species (*Enhalus acoroides, Halodule uninervis, Halophila stipulacea, Thalassia hemprichii and Thalassodendrum ciliatum*). Mean ± SE values are reported. The results of Tukey HSD posthoc tests to assess pairwise differences are indicated: different letters (a,b,c,d) indicate significant differences (*P* < 0.05).

Soil biogeochemical characteristics (dry bulk density, C_org_ content (in % and mg cm^−3^), δ^13^C of soil organic matter, and sediment grain-size composition) differed significantly among study sites and meadows with distinct species composition (*P* < 0.05; Table 4). Soil accretion rates (cm yr^−1^) and C_org_ accumulation rates (g m^−2^ yr^−1^) also differed significantly among locations (*P* < 0.01; Fig. 2A) but did not differ among the seagrass species tested (*P* > 0.05; Fig. 2B), while C_org_ stocks (kg m^−2^ in 1 m-thick soils) did not differ either among locations or meadows with distinctive species composition (*P* > 0.05; Table 4; Fig. 2A,B).

**Table 4:** Results of General Linear Models. Soil dry bulk density, soil accretion rates, organic carbon (C_org_) concentration, stable carbon signatures (δ^13^C) of sedimentary organic matter, and sediment grain size fractions in response to Site (Thuwal Island, Economic City, Petro Rabigh and Khor Alkharar) and Seagrass species (*Halophila stipulacea*, *Thalassia hemprichii*, *Enhalus acoroides*, *Thalassodendrum ciliatum* and *Halodule uninervis*).

Variable	Factor	*F*	d.f.	*P*-value
Dry bulk density (g cm^−3^)	Site	25.771	2	**<0.001**
	Seagrass spp.	17.010	3	**<0.001**
Soil accretion rates (cm yr^−1^)	Site	25.089	2	**<0.001**
	Seagrass spp.	1.153	3	0.360
C_org_ (%)	Site	13.298	2	**<0.001**
	Seagrass spp.	31.146	3	**<0.001**
C_org_ (mg cm^−3^)	Site	7.912	2	**<0.001**
	Seagrass spp.	20.885	3	**<0.001**
C_org_ stock (kg m^−2^ in 1m-thick)	Site	1.375	2	0.276
	Seagrass spp.	1.574	3	0.227
C_org_ accumulation rates (g m^−2^ yr^−1^)	Site	12.721	2	**<0.01**
	Seagrass spp.	2.307	3	0.118
δ^13^C (‰)	Site	241.523	2	**<0.001**
	Seagrass spp.	63.414	3	**<0.001**
<0.063 mm (%)	Site	11.291	2	**<0.001**
	Seagrass spp.	55.494	3	**<0.001**
>0.063 <0.125 mm (%)	Site	169.449	2	**<0.001**
	Seagrass spp.	57.760	3	**<0.001**
>0.125 <0.25 mm (%)	Site	135.947	2	**<0.001**
	Seagrass spp.	34.330	3	**<0.001**
>0.25 <0.5 mm (%)	Site	58.551	2	**<0.001**
	Seagrass spp.	11.601	3	**<0.001**
>0.5 <1 mm (%)	Site	283.002	2	**<0.001**
	Seagrass spp.	72.341	3	**<0.001**

The degrees of freedom (d.f.) for each term in the mixed model analysis are indicated.

Soil dry bulk density in meadows at Economic City (0.9 ± 0.01 g cm^−3^) was lower compared to those at Petro Rabigh and Khor Alkharar (1.1 ± 0.01 g cm^−3^ in both cases), while seagrass soils at Thuwal Island had the highest soil dry bulk density values (1.3 ± 0.01 g cm^−3^). The soil C_org_ content was significantly higher at Economic City (0.4 ± 0.01% C_org_) and Khor Alkharar (0.3 ± 0.01% C_org_) compared to meadows at Thuwal Island (0.2 ± 0.01% C_org_) and Petro Rabigh (0.3 ± 0.03% C_org_; *P* < 0.001; Fig. 2A). At Petro Rabigh, seagrass soil accretion rates (1.3 ± 0.3 cm yr^−1^) and C_org_ accumulation rates (27.3 ± 1.5 g C_org_ m^−2^ yr^−1^) were significantly higher (*P* < 0.001) than those from the other sites (averaging 0.14 ± 0.1 cm yr^−1^ and 4.7 ± 1.0 g C_org_ m^−2^ yr^−1^; Fig. 2A). Soil C_org_ stocks in 1 m-thick deposits were not significantly different among locations (ranging from 2.7 ± 0.15 to 4.0 ± 1.9 kg C_org_ m^−2^).

Seagrass soils at Thuwal Island had higher amounts of fine soil particles (78% of clay and silt and very fine sands) compared to the other locations (ranging from 51 to 63%; Fig. 2A). Meadows at Economic City had a relatively larger proportion of coarse sands (25%) compared to the other locations (ranging from 1 to 14%). Meadows at Thuwal Island and Petro Rabigh were ^13^C-depleted (averaging −17.8 and −22.7‰, respectively) compared to the other locations (δ^13^C ranging from −12.7 to −15.4‰; Fig. 2A). The mixing models applied indicated that seagrass detritus was a relevant source of soil C_org_ in meadows at Economic City (51%) and Khor Alkharar (45%), but a relatively minor contributor at the rest of locations (ranging from 12 to 31%; Fig. 1A). Mangrove plus halophyte constituted the main soil C_org_ sources in Petro Rabigh meadows (73%) and Thuwal Island (38%), while the contribution of seston ranged between 15 and 32% among the study sites.

Dry bulk density was significantly lower in *T. hemprichii* meadows (0.7 ± 0.02 g cm^−3^) compared to the other seagrass species (ranging from 0.9 to 1.3 g cm^−3^). The soil C_org_ content was significantly higher in *T. hemprichii* (0.7 ± 0.02% C_org_) and *E. acoroides* (0.4 ± 0.01% C_org_) compared to *H. stipulacea*, *T. ciliatum* and *H. uninervis* meadows (ranging from 0.2 ± 0.01 to 0.3 ± 0.01% C_org_; Fig. 2B). The soil C_org_ concentration (% and mg C_org_ cm^−3^) decreased down core. Soil C_org_ stocks and accumulation rates were not significantly different among meadows with distinct species compositions (ranging from 2.8 ± 0.4 to 4.8 ± 0.3 kg C_org_ m^−2^ and 5.5 ± 1.3 to 12.47 ± 6.1 g C_org_ m^−2^ yr^−1^, respectively; *P* > 0.05). Seagrass meadows formed by *H. stipulacea*, *T. hemprichii* and *E. acoroides* contained higher amounts of clay and silt particles (ranging from 42 to 48%) compared to those of *T. ciliatum* and *H. uninervis* (ranging from 21 to 22%; Fig. 2B).

Meadows formed by *T. hemprichii*, *E. acoroides* and *H. uninervis* were ^13^C-enriched (ranging from −11.2 to −14‰) compared to *H. stipulacea* (−16.0‰) and *T. ciliatum* (−20.0‰) meadows (Fig. 2B). The trends in δ^13^C values with soil depth remained stable in *T. hemprichii*, *E. acoroides* and *H. uninervis* meadows (Supporting Information Fig. A), indicating that either the organic matter decomposition in the soils or the inputs of organic matter remained stable. However, the δ^13^C values become more negative with depth/ageing in *H, stipulacea* and *T. ciliatum* meadows, in particular below ~60 cm soil depth. The ^13^C-depletion in soil organic matter could either indicate the lack of seagrass matter inputs below cm ~60 in the cores or the decomposition of carbohydrates and proteins with ageing, which have more positive δ^13^C values than the remaining organic matter (e.g. lignin^22^). In order to constrain the uncertainties mentioned above, we run the mixing models using average δ^13^C values within the top 60 cm of the cores only. Despite the δ^13^C values only remained stable within the top 60 cm in *E. acoroides* (R^2^ = 0.05) and *H. stipulacea* (R^2^ = 0.14) meadows (i.e. the δ^13^C values significantly increased with soil depth in *T. hemprichii* meadows (R^2^ = 0.60) and significantly decreased with soil depth in *H. univervis* (R^2^ = 0.30) and *T. ciliatum* (R^2^ = 0.34)), their variability is relatively small within the top 60 cm of soil compared to 1 m-thick soils, thereby constraining the uncertainties associated with diagenetic effects. The mixing models applied indicated that seagrass detritus was a significant source of soil C_org_ in all meadows studied: *T. hemprichii* (58%), *E. acoroides* (59%), *H. uninervis* meadows (45%) and *H. stipulacea* (40%), except *T. ciliatum* (21%; Fig. 1B). Mangrove plus halophyte constituted the main soil C_org_ sources in *T. ciliatum* meadows (54%), while the contribution of seston ranged from 24 to 29% in the meadows among meadows with different species composition.

## Discussion

Seagrass meadows represent one of the most important vegetated communities in the otherwise arid and oligotrophic Red Sea region. Seagrasses are widely distributed along the Kingdom of Saudi Arabia Red Sea coast^18^, and the soils underneath those seagrass meadows contain considerable C_org_ stocks. The soil C_org_ content of Red Sea seagrass in 1 m-thick soils (3.4 kg C_org_ m^−2^ on average) is well below the values from global estimates (ranging from 12 to 83 kg C_org_ m^−2^ ^6^). The relatively low C_org_ sink capacity of Red Sea seagrasses could be due to the extreme environmental conditions such as nutrient limitation and high temperature, reducing the growth rates of the seagrasses and increasing the rate of respiration in the soil^21,23^.

This disconnection between Red Sea seagrass C_org_ stocks and the global estimates are likely linked to the very limited data set used to produce global estimates, which was biased by the extremely high C_org_ content of soils from Mediterranean *Posidonia oceanica* meadows^6^. Recent estimates of soil C_org_ stocks in low biomass seagrass species from Abu Dhabi (*H. uninervis*, *Halophila ovalis* and *H. stipulacea*; ranging from 0.2 to 10.9 kg C_org_ m^−2^ ^24^), Asia (*Zostera* spp. and *T. hemprichii*; ranging from 3.8 to 8.9 kg C_org_ m^−2^ ^20^), and Australia (*H. uninervis* and *T. hemprichii*; ranging from 2.3 to 5.0 kg C_org_ m^−2^ ^10^) are within the range of C_org_ stocks estimated for Red Sea seagrasses. Hence, it is likely that C_org_ stocks in Red Sea seagrass soils tend to be in the lower range but are not necessarily below those in seagrass soils in all other meadows, suggesting a need to update the global estimate of C_org_ content in seagrass soils using a more balanced geographical distribution of seagrass meadows, but also accounting for habitat variability (i.e. diversity of morphological traits across species and geomorphology).

Moreover, long-term C_org_ accumulation rates in Red Sea seagrass (7 g C_org_ m^−2^ yr^−1^ on average) are lower than previous estimates for large and long-living *Posidonia* spp. meadows in the Mediterranean (84 ± 20 g C_org_ m^−2^ yr^−1^) and Australia (12 ± 7 g C_org_ m^−2^ yr^−1^)^25^, but similar to estimates from low biomass and fast-growing seagrass species (i.e. *Zostera* spp. and *T. hemprichii*) from Japan (ranging from 1.8 to 10.1 g C_org_ m^−2^ yr^−1^ ^20^).

Nevertheless, owing to the low C_org_ storage in desert land areas and the relatively large seagrass habitat in the Red Sea coast of Saudi Arabia (370 km^2^ ^18^), seagrasses constitute hotspots of C_org_ storage in this extremely arid region. Multiplying this area by the average C_org_ stocks in 1 m-thick soils and the average C_org_ accumulation rates, yields a total estimate of 1.2 ± 0.1 Tg C_org_ at 1,700 ± 337 Mg C_org_ yr^−1^ in Saudi Arabia meadows. The total C_org_ stored in Saudi Arabia’s seagrass meadows is roughly equivalent to 7 years of total CO_2_ emissions from fossil-fuel burning, cement production, and gas flaring by Saudi Arabia, while seagrass meadows sequester annually around 0.8% of these emissions (Saudi Arabia emissions estimated at 0.16 Tg C at 2014 rates^26^).

Long-term (i.e. based on ^14^C) soil accumulation rates in Red Sea seagrass (ranging from 0.2 to 16 mm yr^−1^; 2.4 mm yr^−1^ on average) are within the range of 14C-derived values reported in previous studies from Australia (ranging from 0.15 to 2.5 mm yr^−1^ ^25,27^), Japan (ranging from 0.37 to 1.3 mm yr^−1^ ^20^), Spain and Italy (ranging from 0.6 to 4.9 mm yr^−1^ ^5,25,28,29^). The capacity of seagrass to elevate the seabed through sediment accretion has been previously recognized as a major component of their role in climate change adaptation^4^, as it helps mitigate against sea level rise. The results obtained in this study confirm that seagrass meadows in the Red Sea play a significant role in climate change adaptation through the protection against sea level rise, despite this being an arid region with very limited supply of terrestrial sediment via run-off. The sea level rise in the coast of Saudi Arabia has been estimated at 2.2 ± 0.5 mm yr^−1 ^^30^, hence seagrass ecosystems along the Saudi Arabian coast have been playing a key role in offsetting relative sea level rise.

**Figure 3:**
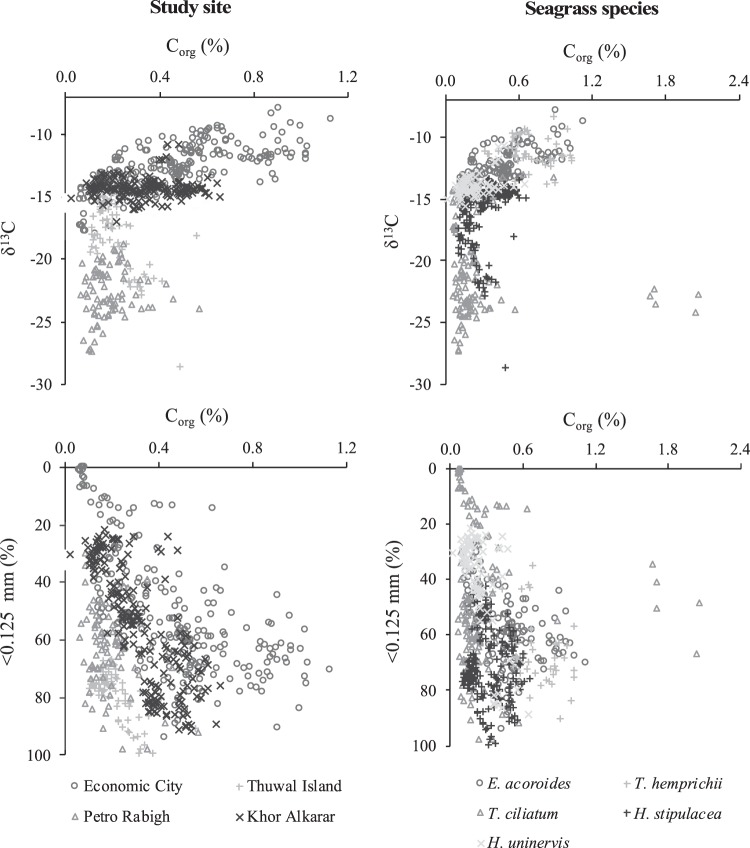
Biplots showing the relationships among the variables studies in the seagrass cores from the Red Sea based on study site and seagrass species. <0.125 mm (%) indicates the percentage of clay and silt and very fine sands within the bulk soil.

The large variability in C_org_ concentrations, stocks and accumulation rates among seagrass habitats (i.e. species composition and location) support the hypothesis that C_org_ storage in seagrass soils is influenced by interactions of biological, chemical and physical factors within the meadow^14,15,31^. Despite that no significant differences in C_org_ stocks among locations existed (at 95% confidence), the biogeochemical characteristics of the cores allowed the reconstruction of the processes and drivers involved in C_org_ storage (Fig. 3A). Soil C_org_ was negatively correlated to soil dry bulk density (R^2^ = 0.34), as previously shown in a range of sediments including seagrass soils^32,33^, which could explain the significant differences found in C_org_ concentration (%) and the lack of differences in C_org_ stocks (kg m^−2^).

The relatively high soil C_org_ concentrations (%) at Economic City could be related to the relatively high accumulation of seagrass detritus and abundance of fine sediments. These results support the hypothesis that the seagrass plants themselves play a key role in determining the amount of C_org_ available for burial^14^, while the presence of fine sediments tends to reduce remineralization rates due to lower oxygen exchange and redox potentials^11,34,35^. The mechanisms behind C_org_ accumulation and preservation in seagrass meadows at Petro Rabigh appear to be mainly related to the relative high soil accumulation rates together with large fluxes of C_org_ from adjacent mangrove and tidal marsh ecosystems. Previous studies have shown that high soil accumulation rates in seagrass meadows, linked to the capacity of their canopy to tap and retain sediment particles^12,36^, the hydrodynamic energy and the production of biogenic carbonates within the meadow^37,38^, contribute to higher accumulation and preservation of C_org_ after burial^14^. Petro Rabigh is an enclosed environment surrounded by mangrove forests, which has been shown to largely contribute to soil C_org_ storage in adjacent seagrass meadows^15,39^. The relatively low soil C_org_ storage of seagrass meadows at Thuwal Island could be explained by the relatively low contribution of seagrass detritus to the soil C_org_ pool and the low soil accumulation rates (Fig. 3A).

Clear differences were observed among meadows with distinct species composition, with the highest soil C_org_ concentrations (%) found in meadows composed of the largest seagrass species *T. hemprichii* and *E. acoroides* (Fig. 3B). However, the relatively low soil dry bulk density found in these meadows led to similar C_org_ stocks among all meadows studied. The results obtained in this study show that soil C_org_ concentration was influenced by the relative contribution of seagrass detritus to the soil C_org_ pool and the amount of fine sediments, which support the results obtained in previous studies^14,15,25^. The relatively high soil C_org_ concentration and seagrass contribution to the soil C_org_ pools in *T. hemprichii* and *E. acoroides* could be explained by the highest above- and below ground biomass of stands formed by these species (ranging from 72 to 87 g DW m^−2^ and 210 to 392 g DW m^−2^, respectively) compared to the other seagrass species studied (ranging from 2.3 to 27 g DW m^−2^ and 2.6 to 61 g DW m^−2^ ^40^. This study supports previous research reporting that the intrinsic properties of the seagrass themselves (e.g. canopy structure, below- and above-ground biomass, and productivity) can influence soil C_org_ storage^14,15,31^. Moreover, the relative constant C stable isotope signatures along the cores confirm the stability of organic sources to soil C_org_ pools, except for *H. stipulacea* and *T. ciliatum* meadows (i.e. δ^13^C values decreased below cm 60), which may indicate that seagrass meadows have only been present for the last centuries at these locations. The presence of coarse soil fibers throughout ~14 cores indicated that seagrasses were present at the coring sites throughout the period reconstructed or the soil depth studied. However, in half of the cores seagrass fibers disappeared at 25–60 cm depth, which could be either due to seagrass absence or the decomposition of coarse organic matter with ageing. Indeed, with the proxies analyzed here it is not possible to assure that the seagrass species occurring at present have remained the same through time.

Our results contribute to gaps in the existing global database on seagrass meadow C_org_ stocks and accumulation rates, which were thus far lacking information from seagrass species in arid environments and suggest that even meadows comprised of ephemeral seagrass species can play an important role in C_org_ sequestration.

## Material and Methods

### Study site and sampling

**Figure 4:**
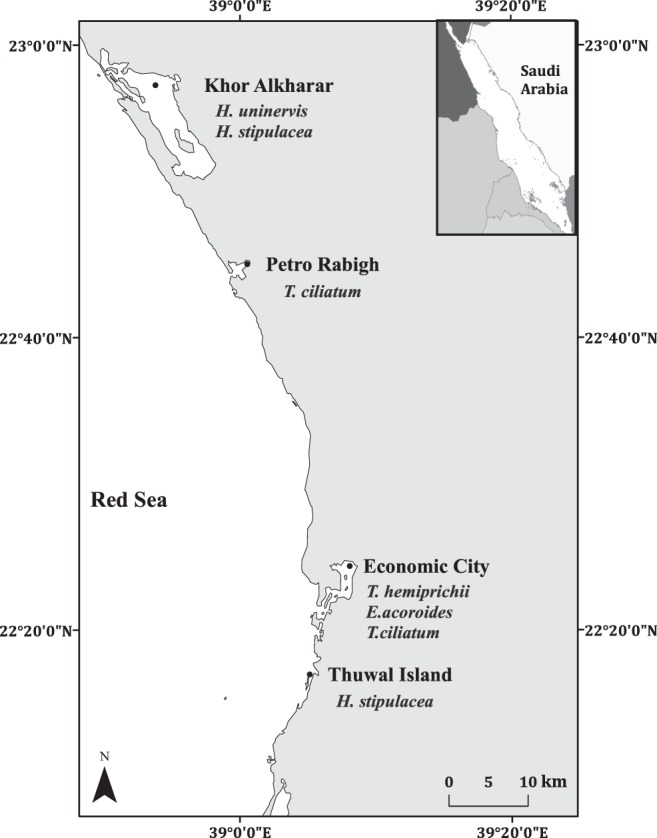
Location of seagrass meadows sampled in Saudi Arabia, Central Red Sea. The map was produced with ArcMap Version 10.2. Background map credits: the World Administrative Divisions layer provided by Esri Data and Maps, and DeLorme Publishing Company. Redistribution rights are granted http://www.esri.com/~/media/Files/Pdfs/legal/pdfs/redist_rights_103.pdf?la=en. The seagrass species present within the meadows surveyed at each study site are indicated.

This study was conducted in four locations (Thuwal Island, Petro Rabigh, Economic City and Khor Alkharar) along 80 km of the Kingdom of Saudi Arabia coastline in the Central Red Sea (Fig. 4). Seagrass meadows are found along the Saudi coast, mainly composed by *H. stipulacea, T. hemprichii, E. acoroides, T. ciliatum and H. uninervis*^18^.

Seagrass meadows at Thuwal Island grow on shallow soil of weathered coral and are located near the fisherman city of Thuwal^41^. Petro Rabigh is a major industrial and petrochemical complex, whereas the Economic City is a newly developed city and harbor complex subject to intense coastal development^41,42^. The Khor Alkharar lagoon encompasses a relatively undeveloped coastal plain and is permanently connected to the Red Sea.

Twenty-seven soil cores were sampled in 1 to 7 m-deep mono-specific seagrass meadows using manual percussion and rotation (PVC pipe with an inner diameter of 60 mm; Supporting Information Table B). Three to four replicate cores were sampled within 100 m^2^ of each mono-specific seagrass meadow at each site (three cores at Thuwal Island, 10 cores at Economic City, four cores at Petro Rabigh and 10 cores at Khor Alkharar). One third of the cores collected at each site were kept inside the PVC corers and transported to the laboratory (hereafter referred to as ‘whole cores’). The other cores from each study site were sampled in the field using a corer consisting of a PVC pipe with pre-drilled holes in the sidewall (3 cm wide and 3 cm apart; hereafter referred to as ‘port cores’), allowing sub-sampling of soil samples along the core in the field by inserting 60 ml syringes into the pre-drilled holes along the PVC pipes (Supporting Information Table B).

The total length of the core barrel used, the empty space inside the barrel before retrieval, the length of barrel outside the soil before retrieval, and the length of retrieved seagrass soil were recorded in order to correct the core lengths for compression effects and all variables studied here are referenced to the corrected, uncompressed depths (Supporting Information Table B). The volume of each subsample retrieved from the port cores was recorded in the field. The whole cores were sealed at both ends, transported vertically and stored at 4 °C before processing in the laboratory.

Plants, seaweeds and seston (>0.7 μm) were sampled across the study sites for isotopic characterization of the potential sources of organic matter in seagrass soils. Seagrasses included *E. acoroides*, *T. hemprichii*, *T. ciliatum*, *H. stipulacea and H. uninervis*. Mangroves and halophytes included *Avicennia marina*, *Salicornia* spp., *Zygophyllum cocenium*, *Anabasis setifera* and *Suaeda monoica*. Seaweeds included *Padina* spp., *Colpomenia sinuosa*, *Sargassum* spp. and *Turbinaria ornata*; seston >0.7 μm. The full dataset and details on methods can be found in Almahasheer *et al*.^17^.

### Laboratory procedures

The whole cores were opened lengthwise and cut into 1 cm-thick slices, and each slice together with the sub-samples from the port cores were oven-dried at 60 °C until constant weight to determine the dry bulk density (g cm^−3^). All samples from the port cores and every second slice of the whole cores were then grounded in an agate mortar and subdivided for analysis.

For the analyses of soil organic carbon (C_org_) and stable isotope composition (δ^13^C), 1 g of ground sample was acidified with 4% HCl to remove inorganic carbon, centrifuged (3400 revolutions per minute, for 5 min), and the supernatant with acid residues was carefully removed by pipette, avoiding resuspension. The sample was then washed with Milli-Q water, centrifuged and the supernatant removed. The residual samples were re-dried and then encapsulated for C analyses using a Thermo Delta V Conflo III coupled to a Costech 4010 at the UH Hilo Analytical Laboratory, USA. The content of C_org_ was calculated for the bulk (pre-acidified) samples. Carbon isotope ratios are expressed as δ values in parts per thousand (‰) relative to the Vienna PeeDee Belemnite standard. Replicate assays and standards indicated measurement errors of 0.01% for C_org_ content and 0.06‰ for δ^13^C.

For sediment grain-size analyses, a Mastersizer 2000-Malvern laser-diffraction particle analyzer was used following sieving (1 mm) and digestion of <1 mm soil samples with 30% hydrogen peroxide. Grain size fractions were categorized following Wentworth scale: clay and silt particles (<0.063 mm), very fine sand (>0.063 <0.125 mm), fine sand (>0.125 <0.25 mm), medium sand (>0.25 <0.5 mm), and coarse sand (>0.5 <0.75 mm)^43^.

A total of 58 radiocarbon analyses were conducted in the 27 cores sampled (1–5 analyses per core) at the AMS Direct Laboratory (USA) following standard procedures^44^. Samples consisted of pooled shells and bulk soil (Supporting Information Table C). Shells were partially digested with 10% HCl, rinsed in ultrapure MQ water in order to remove fine sediment particles, inspected under a stereomicroscope for absence of attached reworked materials, and dried at 60 °C to a constant weight before radiocarbon dating. The ^14^C age-depth models were produced using the R routine “Bacon” for Bayesian chronology building^45^, after ^14^C calibration using the marine13 radiocarbon age calibration curve^46^ taking into account a local Delta R of 110 ± 38 years^47^. From the Bacon routine output, the mean age was used to produce an age-depth weighted regression model forced through 0 (0 cm is cal. BP: 1950), using as weight the sum of the Euclidean distance of the minimum and maximum ages. In four cores, the ^14^C results indicated either that the samples dated were modern (younger than ~400 years) or that the core was mixed (Supporting Information Table C), and therefore we did not produce age-depth models for these four cores. The relatively unknown marine reservoir effects at our study sites (and changes through time) remains a big assumption when calibrating ^14^C ages^48^. All ^14^C results used to model core age-depth chronologies in this study are older than ~400 years, and therefore, the burning of fossil fuels did not affect our ^14^C-derived soil accumulation rates. All dates reported in this paper are expressed as radiocarbon calibrated years.

### Numerical procedures

C_org_ density (g C_org_ cm^−3^) was calculated for each soil depth in each core by multiplying the sediment dry bulk density (g cm^−3^) by the C_org_ concentration (%). For soil depths where C_org_ content (%) was not analyzed, we extrapolated the %C_org_ (i.e. by averaging the %C_org_ between above and below depths) and multiplied the %C_org_ by the dry bulk density (g cm^−3^) to obtain C_org_ density (g C_org_ cm^−3^). To allow direct comparisons among locations, the soil C_org_ standing stocks per unit area (cumulative stocks; kg C_org_ m^−2^) were standardized to 1 m-thick deposits. The total soil depth sampled was higher than 100 cm in 13 cores out of 27 cores sampled and therefore, no extrapolation was required for these cores. However, the soil C_org_ stocks in 1 m-thick soil deposits were inferred in 14 cores (soil depths sampled ranged from 44 to 64 cm) to 1 m, by extrapolating linearly integrated values of C_org_ content (cumulative C_org_ stock; kg C_org_ cm^−2^) with depth. Correlation between extrapolated C_org_ stocks from 44 cm to 1 m and measured C_org_ stocks in 1 m soil cores was r = 0.80 (P < 0.001; Supporting Information Fig. B). Note that scaling C_org_ stocks to 1 m using this method could either lead to over- or underestimates of C_org_ stocks.

Soil accretion rates (expressed in cm yr^−1^), soil accumulation rates (expressed in g DW m^−2^ y^−1^) and soil C_org_ accumulation rates (expressed in g C_org_ m^−2^ y^−1^) for the last millennia were estimated using ^14^C age-depth models (Table 2). Accumulation rates of C_org_ were calculated in 24 out of the 27 cores sampled by multiplying the C_org_ inventories in 1 m-thick soil by the average ^14^C soil accretion rate.

Analyses to test for differences in the variables studied among sites were performed using General Linear Model procedures in SPSS v. 14.0. General Linear Models were used to test for differences in dry bulk density (g cm^−3^), soil accretion rates (cm yr^−1^), soil C_org_ concentration (in %), soil C_org_ density (mg cm^−3^), soil C_org_ stocks (kg m^−2^ in 1 m-thick soils) and soil C_org_ accumulation rates (g m^−2^ yr^−1^), δ^13^C signatures of organic matter, and sediment grain size fractions among study sites and species composition (Table 4), followed by Tukey HSD posthoc tests to assess pairwise differences (Fig. 3). All response variables were square-root transformed prior to analyses and had homogenous variances. Study site (Thuwal Island, Petro Rabigh, Economic City and Khor Alkharar) and seagrass species (*H. stipulacea, T. hemprichii, E. acoroides, T. ciliatum and H. uninervis*) were treated as fixed factors in all statistical models (probability distribution: normal; link function: identity).

Stable Isotope Mixing Models were used to estimate the proportion of the autochthonous and allochthonous C_org_ to the seagrass soil C_org_ pool using δ^13^C and a one-isotope three-source mixing model^49,50^. The δ^13^C signatures within the top 60 cm of each core were pooled and analysed for the probability of relative organic matter contribution to soil stocks using Stable Isotope Mixing Models in R (‘simmr’ and ‘rjags’ packages)^51^. The δ^13^C signatures of potential C_org_ sources (seagrass was considered as autochthonous C_org_, while mangroves plus halophytes, and seaweed plus seston were considered allochthonous C_org_) in the four study sites were obtained from Almahasheer *et al*.^17^. The ‘simmr_mcmc()’ function works by repeatedly producing potential values of the proportional contribution of source material through a Markov chain Monte Carlo, with initial burn-in iterations (1,000) discarded and subsequent iterations (10,000) stored for use in the posterior distribution and analyses of the data^51,52^. Model convergence was confirmed using diagnostic plots and upper confidence intervals, while no overlap between source δ^13^C signature means ± standard deviations were observed. The simmr package allows for the incorporation of δ^13^C uncertainty into mixing models, while producing a Bayesian quantification of the most likely source contributors where there is a greater than n +1 sources when matching against n isotopes^52^. The dataset generated for this manuscript is provided as Supporting Information.

## Electronic supplementary material

Supporting Information

Supporting Dataset
