# Supplementary material for: Carbon stocks and accumulation rates in Red Sea seagrass meadows
Source: Sci Rep. 2018 Oct 9;8:15037. doi: 10.1038/s41598-018-33182-8 (PMC6177483; doi:10.1038/s41598-018-33182-8)

Supporting Information

Carbon stocks and accumulation rates in Red Sea seagrass meadows

Oscar Serrano[1*], Hanan Almahasheer[2], Carlos M. Duarte[3], and Xabier Irigoien[4,5]

[1] School of Science, Centre for Marine Ecosystems Research, Edith Cowan University, 270 Joondalup Drive, Joondalup, Western Australia 6027

[2] Department of Biology, College of Science, Imam Abdulrahman Bin Faisal University (IAU), Dammam 31441-1982, Saudi Arabia

[3] King Abdullah University of Science and Technology (KAUST), Red Sea Research Center, Thuwal 23955-6900, Kingdom of Saudi Araia

[4] AZTI - Marine Research, Herrera Kaia, Portualdea z/g – 20110 Pasaia (Gipuzkoa), Spain

[5] IKERBASQUE, Basque Foundation for Science, Bilbao, Spain

*Corresponding author: o.serranogras@ecu.edu.au

SI Table A. Results of the isotopic mixing model (IsoSource software). Proportion (25%, 50% and 75% quantiles) of autochthonous (i.e. seagrass matter) and allochthonous (i.e. mangroves plus halophytes, seaweed and seston) C_{org} to the seagrass soil C_{org} pool (top 60 cm of the cores). The seagrass species found at each core is indicated.

Core ID	Location	Seagrass species	Autochthonous contribution (%)			Allochthonous contribution (%)					
			Seagrass			Mangroves plus halophytes			Seaweed plus seston		
			25%	50%	75%	25%	50%	75%	25%	50%	75%
T1	Thuwal Island	*H. stipulacea*	15	26	38	35	39	42	20	35	50
T2	Thuwal Island	*H. stipulacea*	14	25	36	37	41	45	19	34	48
T3	Thuwal Island	*H. stipulacea*	31	41	49	31	34	36	16	26	38
EC1	Economic City	*T. hemiprichii*	25	43	61	7	12	17	23	44	66
EC2	Economic City	*T. hemiprichii*	50	62	73	6	10	12	15	28	44
EC3	Economic City	*T. hemiprichii*	63	69	74	13	15	17	9	15	24
EC4	Economic City	*E. acoroides*	44	56	69	6	9	13	18	34	50
EC5	Economic City	*E. acoroides*	33	50	63	6	10	14	22	40	61
EC6	Economic City	*E. acoroides*	51	56	63	20	22	24	14	22	29
EC7	Economic City	*E. acoroides*	62	72	80	5	8	10	11	20	32
EC8	Economic City	*T. ciliatum*	37	48	56	25	28	30	14	24	38
EC9	Economic City	*T. ciliatum*	14	28	43	23	28	32	25	44	62
EC10	Economic City	*T. ciliatum*	13	25	39	28	31	35	25	44	59
PR1	Petro Rabigh	*T. ciliatum*	9	15	20	66	67	69	11	18	25
PR2	Petro Rabigh	*T. ciliatum*	6	10	13	76	78	80	7	12	16
PR3	Petro Rabigh	*T. ciliatum*	6	10	13	76	78	80	7	12	16
PR4	Petro Rabigh	*T. ciliatum*	9	14	20	65	68	71	10	17	24

KA1	Khor Alkharar	*H. uninervis*	15	27	45	14	18	23	32	55	70
KA2	Khor Alkharar	*H. uninervis*	47	53	57	31	32	34	9	15	22
KA3	Khor Alkharar	*H. uninervis*	32	42	51	25	28	31	18	30	43
KA4	Khor Alkharar	*H. stipulacea*	45	53	59	26	29	31	11	18	29
KA5	Khor Alkharar	*H. stipulacea*	37	44	50	33	35	37	13	21	30
KA6	Khor Alkharar	*H. stipulacea*	16	27	40	28	31	35	26	42	56
KA7	Khor Alkharar	*H. stipulacea*	31	37	47	29	31	33	20	32	40
KA8	Khor Alkharar	*H. stipulacea*	48	55	59	27	29	30	10	16	24
KA9	Khor Alkharar	*H. stipulacea*	51	55	59	30	31	32	9	14	18
KA10	Khor Alkharar	*H. uninervis*	55	59	62	30	31	32	6	10	15
**Mean**			**32**	**41**	**50**	**30**	**32**	**35**	**16**	**27**	**38**

SI Table B. Details of the seagrass cores sampled, including the % compression during coring operations. Core length recovered (cm compressed) and core length corrected for compression (cm decompressed).

**core ID**	**Location**	**Seagrass species**	**Water depth (m)**	**Latitude**	**Longitude**	**Corer type**	**Core length (cm compressed)**	**Core length (cm decompressed)**	**Compression (%)**
T1	Thuwal Is.	*H. stipulacea*	1	22° 16.877'	39° 05.120'	Whole	120	131	8
T2	Thuwal Is.	*H. stipulacea*	1	22° 16.867'	39° 05.127'	Port	99	118	16
T3	Thuwal Is.	*H. stipulacea*	1	22° 16.856'	39° 05.108'	Port	117	136	14
EC1	E. city	*T. hemprichii*	0.5	22° 23.652'	39° 07.871'	Port	45	60	25

EC2	E. city	*T. hemprichii*	0.5	22° 23.652'	39° 07.871'	Whole	42	64	34
EC3	E. city	*T. hemprichii*	0.5	22° 23.795'	39° 07.791'	Port	45	50	10
EC4	E. city	*E. acoroides*	0.5	22° 23.781'	39° 07.805'	Port	105	129	19
EC5	E. city	*E. acoroides*	1.5	22° 23.801'	39° 07.836'	Whole	138	158	13
EC6	E. city	*E. acoroides*	2	22° 23.801'	39° 07.836'	Whole	105	116	9
EC7	E. city	*E. acoroides*	0.5	22° 23.766'	39° 07.863'	Port	117	122	4
EC8	E. city	*T. ciliatum*	0.5	22° 22.845'	39° 07.894'	Port	39	44	11
EC9	E. city	*T. ciliatum*	0.5	22° 22.845'	39° 07.894'	Port	39	46	15
EC10	E. city	*T. ciliatum*	0.5	22° 22.845'	39° 07.894'	Whole	50	55	9
PR1	P. Rabigh	*T. ciliatum*	0.5	22° 45.211'	39° 00.681'	Whole	164	206	20
PR2	P. Rabigh	*T. ciliatum*	0.5	22° 45.274'	39° 00.603'	Port	75	113	34
PR3	P. Rabigh	*T. ciliatum*	1	22° 45.197'	39° 00.644'	Port	93	119	22
PR4	P. Rabigh	*T. ciliatum*	1	22° 45.232'	39° 00.645'	Port	87	120	27
KA1	K. Alkharar	*H. uninervis*	0.5	22° 56.025'	38° 52.814'	Whole	120	135	11
KA2	K. Alkharar	*H. uninervis*	1.5	22° 56.105'	38° 52.702'	Port	105	121	13
KA3	K. Alkharar	*H. uninervis*	1.5	22° 56.105'	38° 52.702'	Port	39	47	17
KA4	K. Alkharar	*H. stipulacea*	1.5	22° 56.105'	38° 52.702'	Port	33	47	30
KA5	K. Alkharar	*H. stipulacea*	7	22° 55.886'	38° 53.348'	Port	57	59	3
KA6	K. Alkharar	*H. stipulacea*	7	22° 55.886'	38° 53.348'	Port	51	54	6
KA7	K. Alkharar	*H. stipulacea*	7	22° 55.886'	38° 53.348'	Whole	60	62	3
KA8	K. Alkharar	*H. stipulacea*	3	22° 56.026'	38° 52.888'	Port	57	59	3
KA9	K. Alkharar	*H. stipulacea*	3	22° 56.026'	38° 52.888'	Whole	56	64	13
KA10	K. Alkharar	*H. uninervis*	3	22° 56.026'	38° 52.888'	Port	51	54	6

SI Table C. Radiocarbon results. Cm refers to cm decompressed. The results marked with * were not used to produce the age-depth models (i.e. reversals or modern).

DirectAMS code	Core ID	Depth (cm)	Material	Radiocarbon age BP	1 sigma error
D-AMS 009445	T1	44	Pooled shells	481	20
D-AMS 009446	T1	124	Pooled shells	867	24
*D-AMS 009447	T2	31	Pooled shells	610	25
*D-AMS 009448	T2	104	Pooled shells	530	24
D-AMS 009449	T3	23	Bulk sediment	281	26
D-AMS 009450	T3	135	Bulk sediment	1528	30
*D-AMS 009463	EC1	35	Pooled shells	42	23
D-AMS 009464	EC1	59	Pooled shells	583	25
D-AMS 009477	EC2	30	Pooled shells	895	22
D-AMS 009478	EC2	62	Pooled shells	916	23
*D-AMS 009471	EC3	22	Pooled shells	Modern	
D-AMS 009472	EC3	49	Pooled shells	918	31
D-AMS 009465	EC4	25	Pooled shells	779	26
D-AMS 009466	EC4	128	Pooled shells	1659	25
D-AMS 009555	EC5	23	Pooled shells	662	24
D-AMS 009509	EC5	46	Pooled shells	1222	24
D-AMS 009556	EC5	69	Pooled shells	1743	23
D-AMS 009557	EC5	92	Pooled shells	2006	35
D-AMS 009510	EC5	137	Pooled shells	5458	29
D-AMS 009481	EC6	44	Pooled shells	1239	27
D-AMS 009482	EC6	116	Pooled shells	2730	25

D-AMS 009469	EC7	27	Pooled shells	565	25
D-AMS 009470	EC7	121	Pooled shells	2043	30
D-AMS 009473	EC8	23	Pooled shells	830	22
D-AMS 009474	EC8	43	Pooled shells	1641	23
D-AMS 009475	EC9	24	Pooled shells	979	31
D-AMS 009476	EC9	45	Pooled shells	981	24
D-AMS 009455	EC10	22	Pooled shells	742	26
D-AMS 009456	EC10	54	Pooled shells	799	24
*D-AMS 009526	PR1	50	Pooled shells	2636	25
*D-AMS 009525	PR1	176	Pooled shells	1062	28
*D-AMS 009513	PR2	30	Pooled shells	651	26
*D-AMS 009514	PR2	75		Modern	
*D-AMS 009539	PR3	26	Bulk sediment	Modern	
D-AMS 009540	PR3	118	Pooled shells	253	24
D-AMS 009541	PR4	28	Pooled shells	110	22
D-AMS 009542	PR4	119	Pooled shells	156	25
D-AMS 009521	KA1	45	Pooled shells	1207	21
D-AMS 009522	KA1	135	Pooled shells	1541	25
D-AMS 009497	KA2	23	Pooled shells	1215	25
D-AMS 009498	KA2	120	Pooled shells	1683	27
*D-AMS 009487	KA3	46	Pooled shells	2399	25
*D-AMS 009488	KA3	72	Pooled shells	Modern	
*D-AMS 009485	KA4	28	Pooled shells	Modern	
D-AMS 009486	KA4	46	Pooled shells	1240	24
*D-AMS 009489	KA5	21	Pooled shells	Modern	
D-AMS 009490	KA5	58	Pooled shells	1292	26
*D-AMS 009492	KA6	21	Pooled shells	1089	25
*D-AMS 009491	KA6	53	Pooled shells	715	22

D-AMS 009531	KA7	21	Pooled shells	395	21
D-AMS 009581	KA7	35	Pooled shells	592	35
D-AMS 009532	KA7	61	Pooled shells	806	26
D-AMS 009493	KA8	21	Pooled shells	612	31
D-AMS 009494	KA8	58	Pooled shells	669	24
D-AMS 009527	KA9	23	Pooled shells	490	24
D-AMS 009544	KA9	46	Pooled shells	542	31
D-AMS 009528	KA9	64	Pooled shells	905	22
*D-AMS 009495	KA10	28	Pooled shells	Modern	
D-AMS 009496	KA10	47	Pooled shells	888	24

SI Figure A. Changes in $\delta^{13}C$ values (‰) along soil depth in the *H. stipulacea*, *T. hemprichii*, *E. acoroides*, *T. ciliatum* and *H. uninervis* cores from the Red Sea (average±SE).

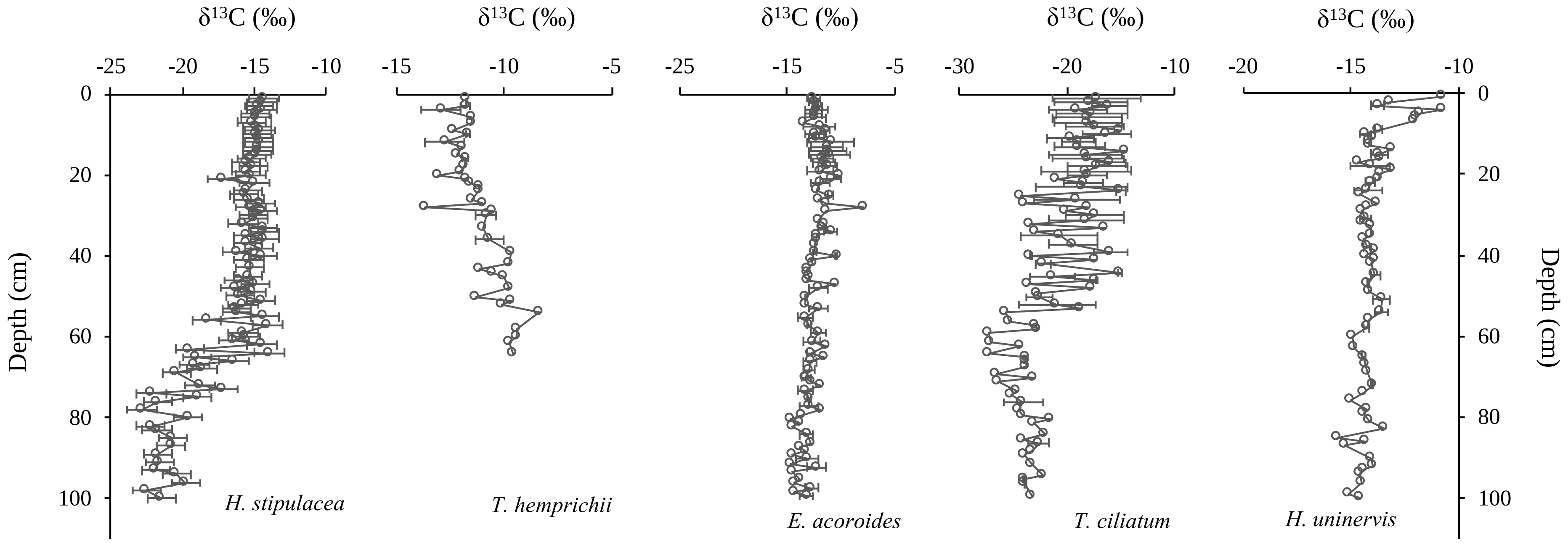

SI Figure B. Relationship between extrapolated and measured C_{org} stocks (a) from 44 cm to 100 cm in seagrass soil cores ≥ 1 m depth.

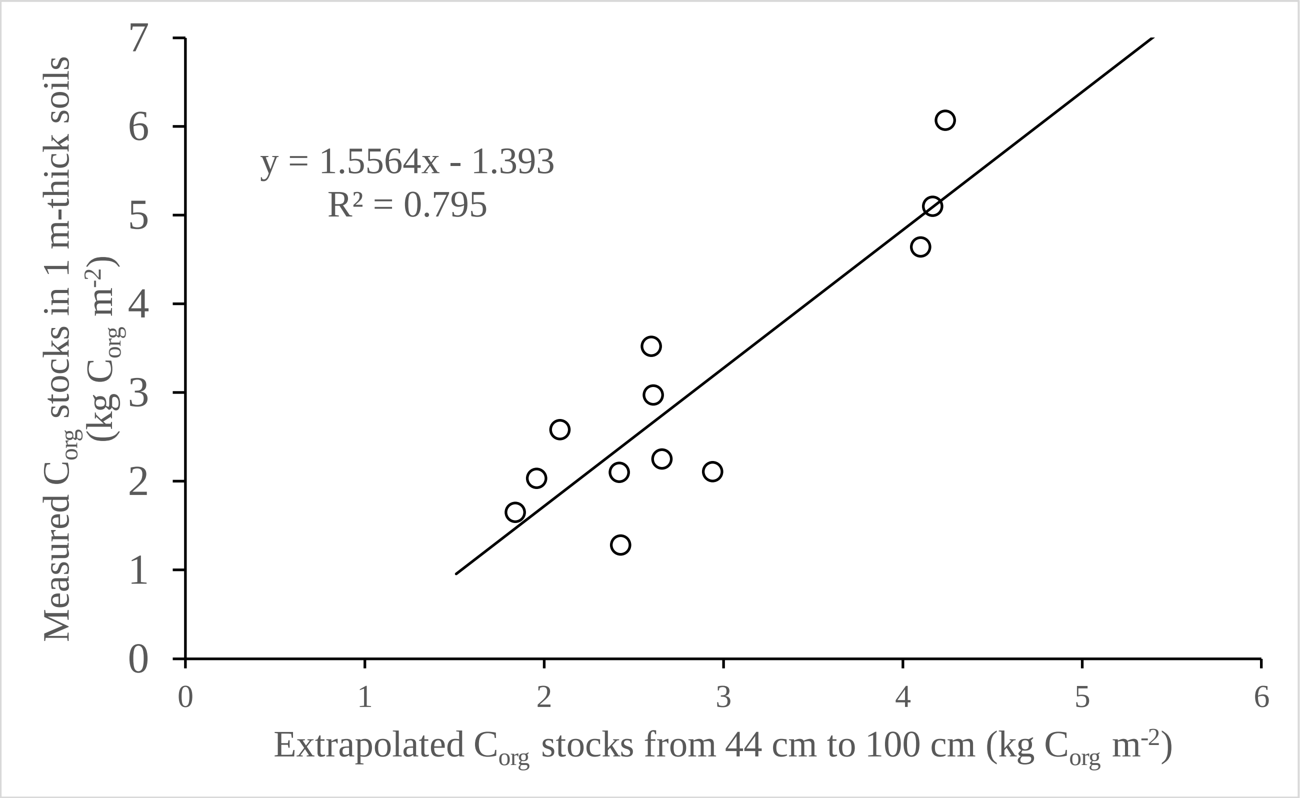

Supplement: Supplementary file 1 — Supporting Information [file 41598_2018_33182_MOESM1_ESM.pdf]
